# QT Dispersion After Primary Percutaneous Coronary Intervention in Patients With ST-Segment Elevation Myocardial Infarction in Relation to Reperfusion and In-Hospital Complications

**DOI:** 10.7759/cureus.112049

**Published:** 2026-07-04

**Authors:** Kyaw Thiri Tun, Khaing Khaing Shein

**Affiliations:** 1 Acute Medicine, North West Anglia NHS Foundation Trust, Peterborough, GBR; 2 Internal Medicine, University of Medicine (1) Yangon, Yangon, MMR; 3 Cardiology, University of Medicine (1) Yangon, Yangon, MMR

**Keywords:** cardiogenic shock, heart failure, left ventricular ejection fraction (lvef), primary percutaneous coronary intervention, qt dispersion, reperfusion, st-segment elevation myocardial infarction

## Abstract

Background: Acute myocardial infarction is one of the leading causes of death worldwide, with mortality primarily resulting from complications such as arrhythmia, cardiogenic shock, and heart failure, which are closely related to the success of reperfusion therapy. Among electrocardiogram (ECG) parameters, the QT interval reflects ventricular homogeneity as well as predicts electrical instability. QT dispersion (QTd), the difference between maximum and minimum corrected QT (QTc) intervals, is associated with fatal cardiac arrhythmia. However, the effect of reperfusion therapy on QTd remains controversial. Although successful reperfusion is a major determinant of outcome in ST-segment elevation myocardial infarction (STEMI), adverse complications such as arrhythmia, cardiogenic shock, and heart failure may still occur despite restoration of coronary blood flow. Currently, no specific marker can reliably predict these adverse events. Assessment of QTd may provide additional prognostic information regarding myocardial electrical recovery following primary percutaneous coronary intervention (PPCI).

Aim: This study aimed to evaluate the association between QTd after PPCI and reperfusion status as well as in-hospital complications among patients with STEMI.

Methods: A total of 108 STEMI patients who underwent PPCI were enrolled. A 12-lead ECG was recorded one hour after PPCI, and QT intervals from 12 leads were manually measured using the slope-intercept method. The QTc interval was calculated using the Fridericia formula. QTd was calculated as the difference between the maximum and minimum QTc intervals.

Results: The mean QTd was 67.68 ± 37.88 milliseconds (ms). QTd was categorized into two groups: >50 ms and ≤50 ms. Reperfusion was measured by ST-segment resolution (STR) and thrombolysis in myocardial infarction (TIMI) flow. In the QTd >50 ms group, 32% had STR ≥70%, while 68% had STR <70%. About 46.6% of the QTd ≤50 ms group had STR ≥70%, whereas 53.4% had STR <70%. This demonstrated that there was no statistically significant association between QTd and STR (p = 0.123). Due to the small sample size and time limitation, only two patients had TIMI 2 flow, whereas the remaining patients had TIMI 3 flow. No statistical significance was observed between QTd and TIMI flow grade (p = 1.000). Cardiogenic shock, heart failure, and reduced ejection fraction (EF) were more frequently observed in the QTd >50 ms group, accounting for 48%, 12%, and 52%, respectively. In the QTd ≤50 ms group, only 5.2% had cardiogenic shock, and 19% had reduced EF but no symptomatic heart failure. There was a significant association between QTd and cardiogenic shock, reduced EF, and symptomatic heart failure with p = 0.001, p = 0.001, and p = 0.008, respectively.

Conclusion: There was a significant association between QTd and cardiogenic shock, heart failure, and an EF of less than 50%. Ventricular tachycardia and post-infarction angina occurred in the QTd >50 ms group, despite the lack of statistical significance due to the limited sample size. QTd may reflect electrical instability of the myocardium and could be a non-invasive tool to identify patients at higher risk of adverse in-hospital complications.

## Introduction

Acute myocardial infarction is a medical emergency in which early diagnosis and timely intervention can improve patient outcomes. In patients with acute ST-segment elevation myocardial infarction (STEMI), primary percutaneous coronary intervention (PPCI) is the preferred reperfusion therapy [1]. Myocardial reperfusion following successful PPCI is commonly assessed using ST-segment resolution (STR) and thrombolysis in myocardial infarction (TIMI) flow grade. However, ventricular repolarization after reperfusion remains controversial, and no definitive method for its assessment has been established [2].

The QT interval represents ventricular systole, the total duration of ventricular depolarization and repolarization. The QT interval duration normally varies between the electrocardiographic leads due to the variation in repolarization and re-excitability among different ventricular regions. Corrected QT (QTc) dispersion is defined as the difference between the maximum and minimum of QTc interval duration [2].

The QTc dispersion is enhanced by the post-myocardial infarction electrophysiological inhomogeneity resulting from an area of necrosis overlapped by ischemic myocardium. These conditions change the electrophysiological properties of the myocardium and lead to regional dispersion [3].

Assessment of myocardial viability can be performed by powerful and expensive tools, such as magnetic resonance imaging, single-photon emission computed tomography, positron emission tomography, and stress electrocardiogram (ECG). However, many authors have addressed QT dispersion (QTd) as a simple, inexpensive, and highly available marker for the evaluation of myocardial viability in their reports [2].

Post-myocardial infarction-related complications including ventricular arrhythmia, cardiogenic shock, and heart failure can lead to significant morbidity like reduced ejection fraction (EF) and wall motion abnormalities. Inadequate reperfusion therapy can be associated with the chance of developing complications, but there have been few indicators to predict complications [1,4].

The QTd has been shown to be a non-invasive indicator of ventricular arrhythmia in acute myocardial infarction, and in one of the studies, left ventricular EF was related to the QTd after PPCI, and the risk of death or severe heart failure was significantly higher in patients with high QTd, providing important prognosis information independently of the degree of STR. These findings suggest that QTd could be an additional tool to reflect not only the status but also the effectiveness of myocardial reperfusion [5].

## Materials and methods

Study design and period

This was a hospital-based prospective cross-sectional analytical study conducted in the Coronary Care Unit of Yangon General Hospital (YGH) in Yangon, Myanmar. All data were collected prospectively over a one-year period from January 2020 to January 2021.

Study population and sample size

**Figure 1:**
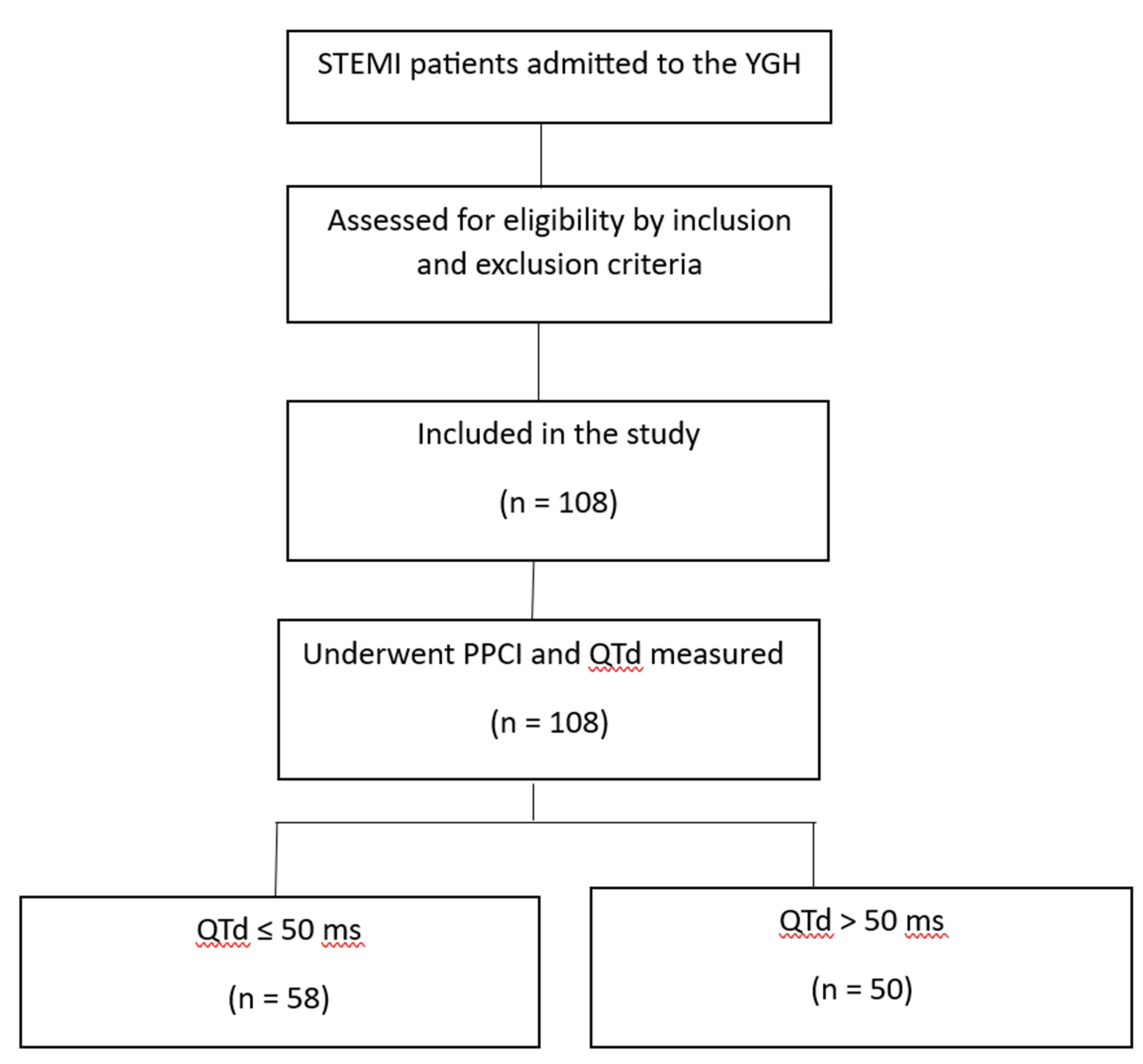
Flow diagram depicting participant enrollment YGH: Yangon General Hospital; STEMI: ST-segment elevation myocardial infarction; QTd: QT dispersion; PPCI: primary percutaneous coronary intervention; ms: milliseconds

A total of 108 patients with acute STEMI who underwent PPCI were enrolled (Figure 1). The minimum required sample size was 105 which was calculated using a standard statistical formula.

The sample size was calculated using the formula $n=\frac{NP(1-P)Z^{2}_{1-&alpha;/2}}{d^{2}(N-1)+P(1-P)Z^{2}_{1-&alpha;/2}}$ [6]. Here, $Z^{2}_{1-&alpha;/2}$ is the standard deviate for delta which is equal to the 95% confidence interval (CI) resulting in 1.96; N is the estimated available study population which is equal to 240 according to the hospital data in 2018; P is the successful reperfusion rate which is equal to 38% [5]; and d is the marginal error which is equal to 0.07 or 7%. Hence, the sample size (n) is equal to 105.

Inclusion and exclusion criteria

Patients were enrolled by using criteria including clinically relevant history suggestive of myocardial infarction, ECG showing ST-segment elevation >1 mm in two adjacent limb leads or >2 mm in >2 adjacent chest leads, and/or elevation of cardiac troponin above the 99th percentile upper reference value [1,4].

Patients with previous myocardial infarction, previous history of thrombolysis, and previous coronary bypass surgery, taking medications that prolong QT interval, and with atrial fibrillation and atrial arrhythmia and electrolyte abnormalities (hypokalemia, hypomagnesemia, hypocalcemia) were excluded from the study because these conditions may affect QT interval measurement and QTd.

Data collection

All patients meeting the inclusion and exclusion criteria were enrolled consecutively, and data were collected using a structured proforma. A standard 12-lead ECG was recorded at a paper speed of 25 mm/s and a calibration of 10 mm/MV amplitude. QTc intervals were measured one hour after PPCI in all leads, and QTd was noted.

ECG analysis

QT interval was measured from the onset of the QRS complex to the end of the T wave defined as the point of return of the T wave to the isoelectric line. QT interval was measured manually by a single observer using a slope-intercept method with the same measurement protocol applied to all patients. QTc interval was calculated using the QTc Fridericia formula [7,8]: $\text{Fridericia formula for QTc}=\mathrm{QT}/\mathrm{RR}^{1/3}$.

QTd is defined as the difference between maximum and minimum QTc values among ECG leads. A QTd of 40-50 milliseconds (ms) was considered normal [9].

The total sum of ST-segment elevation (sSTE) was measured before PPCI and after PPCI. Firstly, the J point was identified, and ST-segment elevation was measured at the J point. STR was calculated as follows: \mathrm{STR}(\%)=[\frac{\text{sSTE before PPCI$-$sSTE after PPCI}}{\text{sSTE before PPCI}}]\times100\%. STR ≥70% was considered adequate resolution [5].

Angiographic finding of PPCI such as TIMI flow grade was noted. In-hospital complications including cardiogenic shock, reduced EF% <50%, heart failure, post-infarct angina, ventricular arrhythmia, and sudden death were observed during hospital stay.

Data analysis

Data from all STEMI patients who underwent PPCI according to the inclusion criteria and exclusion criteria were collected after recording on a proforma. The coding, data entry, and statistical analysis were performed using IBM SPSS Statistics for Windows, V. 20.0 (IBM Corp., Armonk, NY, USA). Descriptive statistics were used to summarize the data. Categorical variables were expressed as frequencies and percentages, while continuous variables were presented as mean ± standard deviation (SD). Associations between QTd groups and reperfusion parameters, including STR and TIMI flow grade, as well as in-hospital complications, were analyzed using the chi-squared test or Fisher's exact test where appropriate. A p-value of <0.05 was considered statistically significant.

Ethical statement

This study was conducted in accordance with the guidelines issued by the Research and Ethics Committee of the University of Medicine (1) Yangon, as part of the institute's Master of Medical Science (MMedSc) in Internal Medicine dissertation program. The research protocol underwent review and approval through the Research and Ethics Committee of the University of Medicine (1) Yangon, on 18th July 2019, prior to the commencement of the study. Data collection was started in January 2020, and patients with STEMI were informed and invited to participate in the study. Daily ECG examination was part of routine clinical care in all admitted STEMI patients and posed no additional risk other than possible minor psychological discomfort. Informed consent was obtained after a thorough explanation of the study design, objectives, procedure, duration of study, and benefits. Participation was voluntary, and participants were free to refuse and withdraw at any time without affecting their medical care or entitlement to benefits. There were no incentives, no extra charges, and no effect on routine procedure by participating in this research. No name was mentioned, and a coded system was used in collecting the data. Confidentiality was protected through the use of coded identifiers, and no personal information was recorded in the study database. Results of this study were used only for research and healthcare purposes.

## Results

The study included 108 patients with a mean age of 56.44 ± 8.65 years. Males accounted for 82.4% of the study population. The mean QTd was 67.68 ± 37.88 ms, ranging from 0 to 172 ms. QTd ≤50 ms was observed in 58 patients (53.7%), whereas 50 patients (46.3%) had QTd >50 ms (Table 1).

**Table 1:** Baseline characteristics of the study population and QTd (N = 108) Data are presented as mean ± standard deviation or n (%). QTd: QT dispersion; ms: milliseconds; n: number of patients

Variable	Value
Mean age, years	56.44 ± 8.65
Male sex, n (%)	89 (82.4)
Female sex, n (%)	19 (17.6)
Mean QTd, ms	67.68 ± 37.88 ms
Minimum QTd, ms	0 ms
Maximum QTd, ms	172 ms
QTd ≤50 ms, n (%)	58 (53.7)
QTd >50 ms, n (%)	50 (46.3)

No statistically significant association was noted between QTd and STR (p = 0.123). Among patients with QTd >50 ms, 68% had STR <70%, whereas 53.4% of patients with QTd ≤50 ms had STR <70% (Table 2).

**Table 2:** Association between QTd and percentage of STR (N = 108) STR: ST-segment resolution; QTd: QT dispersion

QTd	STR ≥70%	STR <70%	Total	χ^2^	P-value
	N (%)	N (%)		2.37	0.123
QTd ≤50 ms	27 (46.6)	31 (53.4)	58 (100)		
QTd >50 ms	16 (32)	34 (68)	50 (100)		
Total	43 (39.8)	65 (60.2)	108 (100)		

The majority of patients in the QTd ≤50 ms group (98.3%) had TIMI flow grade 3, and only 1.7% had TIMI flow grade 2. TIMI flow grade 3 was found in 98% of the QTd >50 ms group, while TIMI flow grade 2 was found in just 2%. There was no significant relationship between QTd and TIMI flow grade when Fisher's exact test was applied (p = 1.000) (Table 3).

**Table 3:** Association between QTd and TIMI flow grade (N = 108) TIMI: thrombolysis in myocardial infarction; QTd: QT dispersion

QTd	TIMI 2	TIMI 3	Total	χ^2^	P-value
	N (%)	N (%)			
QTd ≤50 ms	1 (1.7)	57 (98.3)	58 (100)	0.01	1.000
QTd >50 ms	1 (2)	49 (98)	50 (100)		
Total	2 (1.9)	106 (98.1)	108 (100)		

Among patients with QTd ≤50 ms, only 5.2% developed cardiogenic shock, whereas 48% of patients with QTd >50 ms had cardiogenic shock after PPCI. A statistically significant association was observed between QTd and cardiogenic shock (p < 0.001) by applying the chi-squared test (Table 4).

**Table 4:** Association between the QTd and complications after PPCI (N = 108) QTd: QT dispersion; EF: ejection fraction; PPCI: primary percutaneous coronary intervention

QTd	Cardiogenic shock	Ventricular arrhythmia	Heart failure	EF ≤50%	Post-infarction angina	Death
	N (%)	N (%)	N (%)	N (%)	N (%)	N (%)
QTd ≤50 ms	3 (5.2)	0 (0)	0 (0)	11 (19)	0 (0)	0 (0)
QTd >50 ms	24 (48)	1 (2)	6 (12)	26 (52)	1 (2)	0 (0)
χ^2^	26.27	1.17	7.36	13.01	1.17	-
P-value	<0.001	0.463	0.008	<0.001	0.463	-

QTd was also significantly associated with symptomatic heart failure following PPCI (p = 0.008; Fisher's exact test). Heart failure occurred in 12% of patients with QTd >50 ms, while no symptomatic heart failure was observed in the QTd ≤50 ms group (Table 4).

Reduced left ventricular EF (≤50%) was more frequently observed in patients with QTd >50 ms compared with those with QTd ≤50 ms (52% vs 19%, respectively), demonstrating a statistically significant association (p < 0.001) between increased QTd and impaired left ventricular systolic function (Table 4).

Ventricular arrhythmia and post-infarction angina were noted in the QTd >50 ms group; however, there was no statistical significance due to limited sample size (Table 4).

In summary, patients with QTd >50 ms were more likely to have in-hospital complications, including cardiogenic shock, symptomatic heart failure, and reduced left ventricular EF. However, no significant association was observed between QTd and reperfusion assessed by STR and TIMI flow grade.

## Discussion

The present study evaluated the prognostic significance of QTd in STEMI patients after PPCI. The mean QTd was 67.68 ± 37.88 ms, and the patients were categorized into two groups: ≤50 ms (53.7%) and >50 ms (46.3%). In normal subjects, QTd ranges between 40 and 50 ms; however, a value of 65 ms is regarded by some authors as the upper limit of normal [9]. There is no universally accepted cut-off value for QTd, and the reported upper limit of normal varies among studies depending on the study population and measurement methodology. Although some authors have suggested values up to 65 ms as the upper limit of normal [9], the 50 ms threshold used in the present study was based on previously published literature and large population-based data [10]. Macfarlane et al., in a study of 1,501 apparently healthy adults (863 men and 638 women), reported that a QTd value of 50 ms represented a highly specific upper limit of normal [10]. Accordingly, in the present study, a predefined cut-off value of 50 ms was used to categorize patients into two groups to find out the association with reperfusion and short-term complications after PPCI.

In this study, STR ≥70% was considered indicative of successful reperfusion and was observed in 39.8% of patients. No significant association was found between QTd and the percentage of STR with p = 0.123. These findings are similar to one study involving 153 STEMI patients treated with either PPCI or fibrinolysis. Bao et al. reported that QT metrics were not associated with the severity of ischemia, as quantified by ST-segment elevation. Additionally, relative change in QTc dispersion was weakly associated with reperfusion success assessed by STR at hospital discharge [11].

The findings of the present study, together with previous evidence, suggest that STR may not fully reflect microvascular reperfusion despite being an established marker of myocardial reperfusion. In contrast, QTd may provide additional information regarding myocardial electrical recovery following reperfusion therapy.

In addition to these physiological considerations, the absence of a significant association between QTd and STR may have been influenced by several factors, including variation in ECG acquisition timing after PPCI and the use of a relatively stringent STR cut-off value of 70%. Furthermore, previous studies have used STR cut-off values ranging from 50% to 70% to define reperfusion success [5,12]. In the present study, a cut-off value of STR 70% was used, which may have contributed to statistical insignificance.

In this study, the majority of patients receiving PPCI had TIMI flow grade 3, with only two patients having TIMI 2 (98.1% and 1.9%, respectively). In the QTd ≤50 ms group, the majority of the participants had TIMI 3 (98.3%), whereas only 1.7% had TIMI 2. Similarly, in the QTd >50 ms group, 98% had TIMI 3, while only 2% had TIMI 2. Although the statistical significance cannot be demonstrated, interpretation should be cautious due to limited variability.

According to Fukushima et al., the reduction in QTd following PPCI may be related more to the microvascular reperfusion status than to the location of the infarct-associated artery. Myocardial blush grade (MBG) is an angiographic measure of myocardial perfusion and may remain impaired despite successful PPCI and TIMI 3 flow. The study highlighted that after recanalization, QTd was decreased only in MBG 3 patients, while it remained static in patients with MBG 1 or even rose in those with MBG 2. Successful myocardial tissue reperfusion was associated with the prompt restoration of normal electrical heterogeneity in the myocardium at risk, whereas inadequate tissue reperfusion (MBG 2) was not associated with the resolution of the increased QTd even with TIMI flow grade 3 [13].

Fukushima et al. suggested that QTd may reflect microvascular reperfusion irrespective of TIMI flow grade and restoration of electrical stability which may reduce the risk of subsequent adverse cardiovascular complications [13]. TIMI flow was used to assess reperfusion in this investigation, and TIMI flow reflects the epicardial coronary artery patency rather than the microvascular reperfusion. This may partially account for the absence of an association between QTd and TIMI flow in our study. However, since direct markers of microvascular reperfusion such as MBG, coronary microvascular obstruction, or cardiac magnetic resonance imaging were not evaluated in our study, this explanation remains speculative.

Our study demonstrated that in the QTd >50 ms group, 48% of patients developed cardiogenic shock, 52% had reduced EF ≤50%, 12% had heart failure, and only 2% experienced ventricular arrhythmia and post-infarction angina. These results indicate that patients with QTd >50 ms experienced a higher frequency of in-hospital complications compared to those with QTd ≤50 ms. There was also a significant association between QTd and cardiogenic shock (p < 0.001), reduced EF (p < 0.001), and heart failure (p = 0.008). As only one patient developed ventricular tachycardia and one developed post-infarction angina, statistical significance could not be demonstrated despite both events occurring in the QTd >50 ms group.

Bao et al. stated that prolonged baseline QT metrics were correlated with an increased risk of clinical events with QTc >460 ms and QTc dispersion >65 ms correlated to a >10% risk of developing death, shock, and congestive heart failure. Although caution must be employed when using arbitrary cut points, a baseline QTc dispersion beyond an upper boundary of 65 ms appears to denote significant risk of adverse hospital events [11].

The association between increased QTd and adverse cardiovascular outcomes observed in the present study is consistent with previous reports [11] suggesting that QTd is associated with myocardial electrical instability following STEMI. However, due to the observational nature of this study, these findings should be viewed as associations rather than proof of causation. Persistent electrical heterogeneity may contribute to impaired ventricular function, cardiogenic shock, and heart failure after STEMI. However, given the observational nature of this study, increased QTd may also simply represent as a marker of more extensive myocardial injury, larger infarct burden, or worse ventricular dysfunction rather than an independent causal factor. Therefore, although QTd may provide a simple, inexpensive, and non-invasive parameter for identifying patients at higher risk of adverse in-hospital outcomes after PPCI, larger prospective studies with adjustment for potential confounding factors are required before its independent prognostic value and clinical utility can be established, particularly in resource-limited settings.

The present findings, interpreted in the context of previous published study [12], support the hypothesis that microvascular reperfusion may play an important role in the restoration of myocardial electrical stability, which may not be fully captured by TIMI flow grade and STR. These findings support the hypothesis that QTd may provide additional information regarding myocardial electrical recovery following PPCI and may reflect aspects of myocardial reperfusion. Clinically, this is important because increased post-procedural QTd was significantly associated with cardiogenic shock, reduced EF, and heart failure, suggesting a potential role for QTd in the early identification of patients at higher risk of adverse outcomes.

Limitations

This study has several limitations. It was a single-centre study with a relatively small sample size. The small number of patients exhibiting TIMI 2 flow after PPCI limited meaningful comparison and association between TIMI flow grades. Only short-term in-hospital outcomes were evaluated, with no long-term follow-up data available to assess sustained clinical effects. QTd was measured manually by a single observer. Although this eliminated interobserver variability, intraobserver variability was not formally assessed. Due to the small sample size and low frequency of adverse outcomes, multivariable analysis was not performed, and the observed associations should be considered exploratory rather than evidence of an independent predictive relationship. In addition, several potentially important confounding variables, including infarct location, culprit vessel distribution, ischemic time, symptom-to-balloon time, medication use, and baseline left ventricular function, were unavailable for adjustment. While QTc intervals were measured during the manual calculation of QTd, the individual QTc values were not retained in the original dataset. Consequently, the relationship between QTc prolongation and adverse clinical outcomes could not be evaluated, limiting the completeness of the electrophysiological assessment. Larger prospective multicentre studies with more comprehensive clinical data are needed to validate these findings.

## Conclusions

Prolonged QTd after PPCI emerged in this study as a potential non-invasive marker associated with adverse clinical outcomes in STEMI patients. Patients with QTd >50 ms had significantly higher rates of cardiogenic shock, heart failure, and reduced EF. These findings suggest that QTd may reflect myocardial electrical instability and aspects of microvascular reperfusion that are not fully represented by conventional reperfusion markers alone. However, since direct markers of microvascular reperfusion, including MBG and cardiac magnetic resonance findings, were not evaluated in the present study, this hypothesis remains speculative and should not be interpreted as direct evidence from the present study.

Although no significant association was observed between QTd and STR, comparison according to TIMI flow grade was limited because nearly all patients achieved TIMI 3 flow, reducing the ability to detect a meaningful association. Ventricular tachycardia and post-infarction angina were observed only in patients with prolonged QTd, although statistical significance could not be demonstrated because of the small number of events.

As this was a single-centre study with a relatively small sample size, further multicentre prospective studies incorporating serial ECG assessment and larger high-risk populations are needed to validate the prognostic value of QTd after PPCI and to determine its potential role in early risk stratification and the prediction of post-infarction complications in STEMI patients.

## References

[1] Byrne RA, Rossello X, Coughlan JJ (2023). 2023 ESC guidelines for the management of acute coronary syndromes: developed by the task force on the management of acute coronary syndromes of the European Society of Cardiology (ESC). Eur Heart J.

[2] Tayyebi M, Eshraghi A, Alizadeh Z, Moravveji far K (2016). QT dispersion as a prognostic indicator for myocardial viability: a systematic review. J Patient Saf Qual Improve.

[3] Bhawani G, Kumar A, Murthy KS, Kumari N (2013). Study of QT interval dispersion in acute myocardial infarction and its relationship with complications. J Pharm Biomed Sci.

[4] Thygesen K, Alpert JS, Jaffe AS, Chaitman BR, Bax JJ, Morrow DA, White HD (2018). Fourth universal definition of myocardial infarction (2018). Circulation.

[5] Jiménez-Candil J, Hernández Hernández J, Aguero VL, Martín A, Martín F, Moríñigo JL, Martín-Luengo C (2009). Early reduction of QT dispersion after primary percutaneous intervention in ST-segment elevation acute myocardial infarction. Mechanisms and clinical implications. Cardiology.

[6] Wayne WD (1995). Biostatistics, A Foundation for Analysis in Health Sciences. Biostatistics: A Foundation for Analysis in Health Sciences.

[7] Sagie A, Larson MG, Goldberg RJ, Bengtson JR, Levy D (1992). An improved method for adjusting the QT interval for heart rate (the Framingham Heart Study). Am J Cardiol.

[8] Luo S, Michler K, Johnston P, Macfarlane PW (2004). A comparison of commonly used QT correction formulae: the effect of heart rate on the QTc of normal ECGs. J Electrocardiol.

[9] Antzelevitch C, Shimizu W, Yan GX (1999). The M cell: its contribution to the ECG and to normal and abnormal electrical function of the heart. J Cardiovasc Electrophysiol.

[10] Macfarlane PW, McLaughlin SC, Rodger JC (1998). Influence of lead selection and population on automated measurement of QT dispersion. Circulation.

[11] Bao MH, Armstrong PW, Zheng Y, Westerhout CM, Siha H, Welsh RC (2014). The prognostic relationship of QT interval and dispersion in patients with acute ST elevation myocardial infarction. Exp Clin Cardiol.

[12] Buller CE, Fu Y, Mahaffey KW (2008). ST-segment recovery and outcome after primary percutaneous coronary intervention for ST-elevation myocardial infarction: insights from the Assessment of Pexelizumab in Acute Myocardial Infarction (APEX-AMI) trial. Circulation.

[13] Fukushima N, Tsurumi Y, Jujo K (2014). Impact of myocardial reperfusion status on QT dispersion after successful recanalization of the infarct-related artery in acute myocardial infarction. J Interv Cardiol.

